# PD-L1 and tumor-infiltrating CD8^+^ lymphocytes are correlated with clinical characteristics in pediatric and adolescent pituitary adenomas

**DOI:** 10.3389/fendo.2023.1151714

**Published:** 2023-06-22

**Authors:** Mengwu Shi, Yifu Song, Yaochuan Zhang, Longjie Li, Juanhan Yu, Ana Hou, Sheng Han

**Affiliations:** ^1^ Department of Neurosurgery, The First Hospital of China Medical University, Shenyang, China; ^2^ Department of Pathology, First Affiliated Hospital and College of Basic Medical Sciences, China Medical University, Shenyang, China; ^3^ Department of Pediatrics, Shengjing Hospital of China Medical University, Shenyang, China

**Keywords:** pediatric, adolescent, CD8^+^ TILs, PD-L1, tumor microenvironment, pituitary adenoma

## Abstract

**Objective:**

To investigate the levels of tumor-infiltrating CD8^+^ lymphocytes (CD8^+^ TILs) and the expression of programmed cell death receptor ligand 1 (PD-L1) in the tumor microenvironment (TME) of pediatric and adolescent pituitary adenomas (PAPAs) and analyze the correlation between their levels and the clinical characteristics.

**Methods:**

A series of 43 PAPAs cases were enrolled over a period of 5 years. To compare the TME of PAPAs and adult PAs, 43 PAPAs cases were matched with 60 adult PAs cases (30 cases were between 20 and 40 years old, and 30 cases were older than 40 years) for main clinical characteristics. The expression of immune markers in PAPAs was detected by immunohistochemistry, and their correlation with the clinical outcomes was analyzed using statistical methods.

**Results:**

In the PAPAs group, CD8^+^ TILs level was significantly lower (3.4 (5.7) vs. 6.1 (8.5), p = 0.001), and PD-L1 expression (0.040 (0.022) vs. 0.024 (0.024), p < 0.0001) was significantly higher as compared with the older group. The level of CD8^+^ TILs was negatively correlated with the expression of PD-L1 (r = −0.312, p = 0.042). Moreover, CD8^+^ TILs and PD-L1 levels were associated with Hardy (CD8, p = 0.014; PD-L1, p = 0.018) and Knosp (CD8, p = 0.02; PD-L1, p = 0.017) classification. CD8^+^ TILs level was associated with high-risk adenomas (p = 0.015), and it was associated with the recurrence of PAPAs (HR = 0.047, 95% CI 0.003–0.632, p = 0.021).

**Conclusion:**

Compared with the TME in adult PAs, the TME in PAPAs was found to express a significantly altered level of CD8^+^ TILs and PD-L1. In PAPAs, CD8^+^ TILs and PD-L1 levels were associated with clinical characteristics.

## Introduction

Pituitary adenomas (PAs) are adenohypophysis-derived neoplasms that are the third most prevalent intracranial tumors, with a global incidence of 3.9–7.4/100,000 (1). PAs are, however, quite rare in pediatric and adolescent patients (age ≤ 20), accounting for only approximately 2.6%–6.1% of all pituitary adenoma patients (2, 3). Despite the unique clinical and pathological characteristics of pediatric and adolescent pituitary adenomas (PAPAs), their oncogenic mechanisms are poorly understood, owing to the rare nature of PAPAs (2, 4). Compared to adult PAs, PAPAs vary greatly in their subtypes, tumor size, age of onset of symptoms, and other clinical features (2, 5). In addition, some high-risk adenomas (6) and PAs with extensive invasion, especially those with cavernous sinus involvement, are therapeutically challenging, and new treatment strategies including immunotherapy are being studied.

The tumor microenvironment (TME) of both benign and malignant tumors has received increasing attention, and immunotherapies based on the TME have been shown to provide significant clinical benefits (7). Investigation of the immune microenvironment in intracranial tumors has demonstrated that tumor-infiltrating CD8^+^ lymphocytes (CD8^+^ TILs) play a critical role in the TME of brain tumors (8). It has been reported that CD8^+^ TILs are found in the TME of PAs (9–12). In several human malignancies, CD8^+^ TILs have been shown to improve patient survival (13–15). However, the function of CD8^+^ TILs in PAPAs remains unclear. As an inhibitory immune checkpoint protein, programmed cell death receptor ligand 1 (PD-L1) is predominantly expressed on tumor cells and antigen-presenting cells and is known to inhibit the killing effect of T cells on tumor cells (16). Although the expression of PD-L1 in PAs has been reported by several studies (10, 17, 18), its role in PAPAs is still largely unknown. Immune-checkpoint inhibitors (ICIs) such as anti-PD-L1 therapies have shown great prospects in several human malignancies (19–21).

In the present study, a series of 43 PAPAs cases over a period of 5 years were enrolled. To compare the TME of PAPAs and adult PAs, 43 PAPAs cases were matched with 60 adult PAs cases. Our first objective was therefore to evaluate the expression of CD8^+^ TILs and PD-L1 in patients with PAs across different age groups. Our second aim was to explore the correlation between the above two biomarkers and clinical characteristics in PAPAs.

## Methods

### Patients

**Figure 1:**
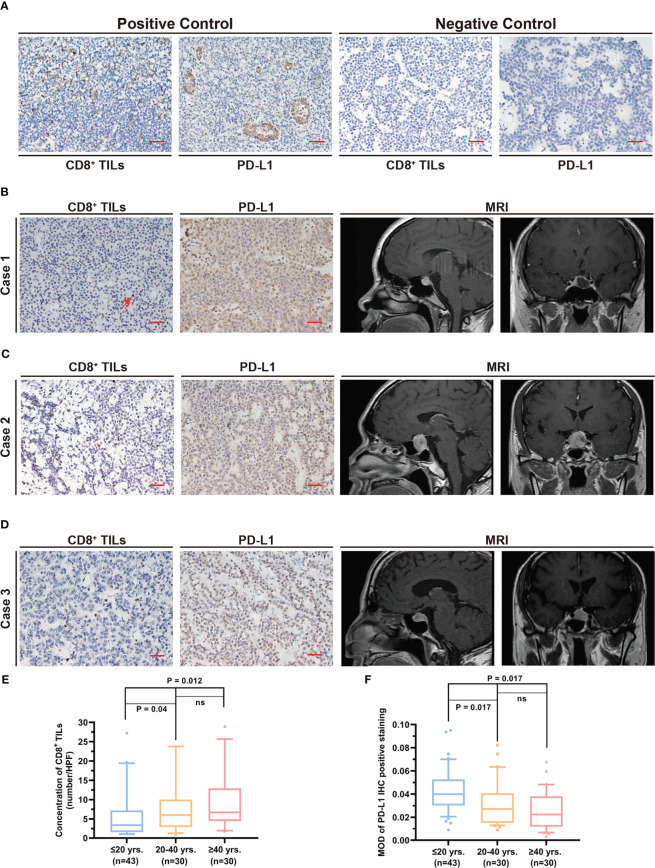
Quantification of the level CD8^+^ TILs and PD-L1 expression in different age groups. **(A)** Positive and negative controls for CD8^+^ TILs and PD-L1 staining. Scale bar, 20 µm. **(B–D)** Typical CD8^+^ TILs and PD-L1 staining and preoperative MRI. Scale bar, 20 µm. **(B)** The patient was 15 years old. The red arrow indicates CD8^+^ TILs positive staining. **(C)** The patient was 35 years old. **(D)** The patient was 64 years old. **(E)** CD8^+^ TILs level in PAPAs group was significantly lower, while no statistical difference between the other two age groups was observed. **(F)** PD-L1 expression in PAPAs group was significantly higher, while no statistical difference between the other age groups was observed. TILs, tumor-infiltrating lymphocytes; PAPAs, pediatric and adolescent pituitary adenomas.

Between January 2012 and December 2016, a total of 1,375 PAs patients underwent trans-sphenoidal surgery at The First Hospital of China Medical University’s Neurosurgery Department. Among them, 43 (3.13%) patients were under the age of 20, and 1,332 (96.87%) patients were older than 20 years. All cases under the age of 20 were enrolled. For comparison, 43 PAPAs cases were matched with 60 adult PAs cases (30 cases were 20–40 years old, and 30 cases were older than 40 years) for sex, main clinical symptoms, preoperative dysfunction, subtypes, high-risk adenomas (2017), maximum diameter, tumor resection degree, PA apoplexy, Hardy and Knosp classification, and tumor recurrence (**Table 1**). Patients with incomplete data were excluded. The above procedure was performed under the direction of an epidemiologist and a statistician. Tumor subtypes were classified according to the 2017 World Health Organization (WHO) classification of pituitary tumors (6). In addition, PAs were categorized into microadenomas (<10 mm), macroadenomas (1–4 cm), and giant adenomas (>4 cm) based on maximum tumor diameter (22). None of the patients had other intracranial tumors, cancers, or systemic diseases. All patients had complete follow-up data. The First Hospital of China Medical University institutional review board approved the present study (No. 2022098), and each patient or their parents provided signed informed consent. Sellar region magnetic resonance imaging (MRI) was regularly performed on all patients (**Figures 1B–D**).

**Table 1:** Clinical features of pituitary adenomas cases.

Age groups	≤20 years	20–40 years	>40 years	>20 years	Total	p	p
	(n = 43)	(n = 30)	(n = 30)	(n = 60)	(n = 103)		
**Age (years, median (IQR))**	18.0 (3.0)	32.5 (8.5)	63.0 (12.8)	40.5 (30.8)	29.0 (30.0)	<0.0001	
Sex							0.988
Male (no. %)	20 (46.5%)	14 (46.7%)	14 (46.7%)	28 (46.7%)	48 (46.6%)		
Female (no. %)	23 (53.5%)	16 (53.3%)	16 (53.3%)	32 (53.3%)	55 (53.4%)		
**CD8^+^ TILs (no. median (IQR))**	3.4 (5.7)	6.0 (7.1)	6.7 (8.4)	6.1 (8.5)	5.2 (6.6)	0.001	
**PD-L1 (MOD, median (IQR))**	0.040 (0.022)	0.027 (0.026)	0.023 (0.026)	0.024 (0.024)	0.031 (0.024)	<0.0001	
Main clinical symptoms (no. %)							0.849
With	33 (76.7%)	24 (80.0%)	23 (76.7%)	47 (78.3%)	80 (77.7%)		
Without	10 (23.3%)	6 (20.0%)	7 (23.3%)	13 (21.7%)	23 (22.3%)		
Preoperative dysfunction (no. %)							0.993
With	33 (76.7%)	23 (76.7%)	23 (76.7%)	46 (76.7%)	79 (76.7%)		
Without	10 (23.3%)	7 (23.3%)	7 (23.3%)	14 (23.3%)	24 (23.3%)		
Pituitary adenoma type (no. %)							0.121
Lactotroph adenoma	23 (53.5%)	10 (33.3%)	10 (33.3%)	20 (33.3%)	43 (41.7%)		
Corticotroph adenoma	5 (11.6%)	5 (16.7%)	9 (30.0%)	14 (23.3%)	19 (18.4%)		
Somatotroph adenoma	4 (9.3%)	8 (26.7%)	3 (10.0%)	11 (18.3%)	15 (14.6%)		
Non-functional adenoma	11 (25.6%)	7 (23.3%)	8 (26.7%)	15 (25.0%)	26 (25.2%)		
High-risk adenomas (no. %)							0.947
Yes	11 (25.6%)	7 (23.3%)	8 (26.7%)	15 (25.0%)	26 (25.2%)		
No	32 (74.4%)	23 (76.7%)	22 (73.3%)	45 (75.0%)	77 (74.8%)		
Classification based on size (no. %)							0.885
Microadenomas (<1 cm)	2 (4.7%)	3 (10.0%)	0	3 (5.0%)	5 (4.9%)		
Macroadenomas (1–4 cm)	37 (86.0%)	24 (80.0%)	29 (96.7%)	53 (88.3%)	90 (87.4%)		
Giant adenomas (>4 cm)	4 (9.3%)	3 (10.0%)	1 (3.3%)	4 (6.7%)	8 (7.7%)		
Resection degree							0.798
NTR (no. %)	35 (81.4%)	25 (83.3%)	25 (83.3%)	50 (83.3%)	85 (82.5%)		
STR (no. %)	8 (18.6%)	5 (16.7%)	5 (16.7%)	10 (16.7%)	18 (17.5%)		
Pituitary adenoma apoplexy (no. %)							0.934
Yes	29 (67.4%)	20 (67.7%)	20 (67.7%)	40 (67.7%)	69 (67.0%)		
No	14 (32.6%)	10 (33.3%)	10 (33.3%)	20 (33.3%)	34 (33.0%)		
Hardy classification (no. %)							0.87
Grades A, B	28 (65.1%)	19 (63.3%)	21 (70.0%)	40 (66.7%)	68 (66.0%)		
Grades C, D, E	15 (34.9%)	11 (36.7%)	9 (30.0%)	20 (33.3%)	35 (34.0%)		
Knosp classification (no. %)							0.834
Grades 0, 1, 2	30 (69.8%)	23 (76.7%)	20 (66.7%)	43 (71.7%)	73 (70.9%)		
Grades 3, 4	13 (30.2%)	7 (23.3%)	10 (33.3%)	17 (28.3%)	30 (29.1%)		
Recurrence (no. %)							0.972
Yes	8 (18.6%)	6 (20.0%)	5 (16.7%)	11 (18.3%)	19 (18.4%)		
No	35 (81.4%)	24 (80.0%)	25 (83.3%)	49 (81.7%)	84 (81.6%)		

p-Values were used to show the statistical difference between the age group younger than 20 years and the age group older than 20 years.

IQR, interquartile range; NTR, near total resection; STR, subtotal resection; MOD, mean optical density; TILs, tumor-infiltrating lymphocytes.

### Immunohistochemistry

Immunohistochemistry (IHC) analysis was performed as per the previous protocol (23, 24) and was conducted according to the manufacturer’s instructions (anti-CD8, Proteintech, Wuhan, China, Cat No. 66868-1-Ig, 1:4,000; anti-PD-L1, Proteintech, China, Cat No. 66248-1-Ig, 1:500). Three paraffin-embedded pituitary adenoma sections were generated from each case through serial sections. Briefly, antigen retrieval was accomplished in sodium citrate buffer (pH 6.0) by microwave after deparaffinization with xylene. Non-specific staining and endogenous peroxidase on sections were blocked, followed by incubating sections with the primary antibody against CD8 and PD-L1. A negative control was performed with normal mouse serum (**Figure 1A**). Further, the sections were treated with the secondary antibody, which was conjugated with horseradish peroxidase, stained with diaminobenzidine, and counterstained with hematoxylin. Human tonsil tissue was used as the positive control for immunohistochemical staining (**Figure 1A**).

### Immunohistochemical quantification

The quantification process was performed by two neuropathologists. A light microscope (Olympus, Tokyo, Japan) was used to observe and photograph the IHC staining results. Images with poor staining or tissue integrity were excluded. Each section was examined using at least five separate high-power fields (HPF, ×400), and the average count for subsequent analysis was used. ImageJ software (http://imagej.nih.gov/ij/) was used for the quantification of IHC positive staining. CD8^+^ TILs were quantified by direct cell count. The mean optical density (MOD) value was used for statistical analysis of PD-L1 expression (24). Subsequently, two neuropathologists performed manual counting of IHC staining results to exclude non-specific staining, and the results were consistent with the computerized analysis.

### RT-qPCR

Resected tumor tissues from our study group of patients who underwent surgical resection were frozen and preserved. Total RNA was extracted from frozen tissues using the TRIzol reagent. Quantitative RT-PCR (RT-qPCR) analyses were performed to detect human *CD8* and *PD-L1* mRNA levels by SYBR Green Real-Time PCR Master Mixes (Applied Biosystems, Foster City, CA, USA). Human *GAPDH* was used as an internal control. The primers are listed in the **Supplementary Material Table**.

### Statistical analyses

The results were presented as the median (interquartile range (IQR)). The Mann–Whitney test, Kruskal–Wallis test, analysis of variance, chi-square test, and Student’s t-test were used to assess statistical significance. Linear correlation was used to assess the correlation between IHC results and the clinical features. Cox proportional hazards regression model analysis was employed to calculate the hazard ratio (HR) of recurrence. The Kaplan–Meier analysis and log-rank test were used to evaluate the difference in recurrence. Appropriate statistical methods were applied depending on whether the data coincided with normal distribution. p < 0.05 (two-tailed) was considered to be statistically significant. All statistical analyses were performed by GraphPad Prism 9 (GraphPad Software Inc., La Jolla, CA, USA) and SPSS v26.0 (SPSS Inc., Chicago, IL, USA).

## Results

### Clinical features

The clinical information of all enrolled cases is presented in **Table 1**. Among the 43 PAPAs patients, there were 20 (46.5%) men and 23 (53.5%) women, with an average age of 18.0 (3.0) years. Before surgery, the main clinical symptoms were headache (n = 18, 41.9%), visual field defect (n = 11, 25.6%), temporal hemianopsia (n = 5, 11.6%), and galactorrhea–amenorrhea (n = 14, 32.6%). There were 33 (76.7%) patients who had pre-operative hypopituitarism. There were 23 (53.5%) patients with lactotroph adenoma, 5 (11.6%) patients with corticotroph adenoma, 4 (9.3%) patients with somatotroph adenoma, and 11 (25.6%) patients with non-functional adenomas. There were 11 (25.6%) patients with high-risk adenomas. Data from multiple centers have revealed that lactotroph adenoma is predominant among pediatric PAs, accounting for 39%–72.7% of all cases (25–27). In the published series of pediatric PAs patients who underwent surgery, lactotroph adenoma also accounts for the vast majority, approximately 47.1%–62% of all cases (27–29). Our indications for lactotroph adenoma surgery included the following: 1) patients with neurosurgical emergencies, 2) patients with pituitary apoplexy, and 3) patients who were intolerant to dopamine agonists or did not have an obvious alleviation or were reluctant to take medication but preferred surgery. Our guideline was consistent with that of other centers (27–29). The median maximum diameter of the tumors was 2.4 (1.6) cm. Two (4.7%) patients had microadenomas, 37 (86.0%) patients had macroadenomas, and 4 (9.3%) patients had giant adenomas. Thirty-five (81.4%) patients experienced tumor near total resection (NTR), and 8 (18.6%) patients experienced tumor subtotal resection (STR). Twenty-nine (67.4%) patients had PA apoplexy. The Hardy and Knosp classification was utilized to classify tumor invasion into the suprasellar and parasellar regions, respectively. Twenty-eight (65.1%) patients were Hardy A/B grade, while 15 (34.9%) patients were Hardy C/D/E grade. Thirty (69.8%) patients were Knosp 0/1/2 grade, while 13 (30.2%) patients were Knosp 3/4 grade. After a median follow-up of 75.0 (29.0) months, 8 (18.6%) patients experienced recurrent tumors, and the median progression-free survival (PFS) was 72.0 (29.0) months.

### The CD8^+^ TILs levels and PD-L1 expression in the different age groups

The levels of CD8^+^ TILs and PD-L1 are shown in **Table 2**. The number of CD8^+^ TILs in the pediatric and adolescent group (3.4 (5.7)) was significantly lower than that of the >20 years group (6.1 (8.5), p = 0.001). Furthermore, after Bonferroni correction, the CD8^+^ TILs level in the pediatric and adolescent group was significantly lower than that of the 21–40 years group (6.0 (7.1), p = 0.04) and >40 years group (6.7 (8.4), p = 0.012). However, the 21–40 years groups and >40 years group did not show statistically significant differences in CD8^+^ TILs levels (p = 1.00; **Figures 1B–E**).

**Table 2:** Clinical and IHC results of pituitary adenomas.

	CD8^+^ TILs (no. median (IQR))			PD-L1 (MOD, median (IQR))		
	≤20 years	>20 years	p	≤20 years	>20 years	p
**Total**	3.4 (5.7)	6.1 (8.5)	0.001	0.040 (0.022)	0.024 (0.024)	<0.0001
Sex						
Male	2.7 (5.9)	6.0 (8.1)	0.013	0.041 (0.019)	0.029 (0.023)	0.008
Female	3.8 (5.8)	6.4 (9.3)	0.03	0.036 (0.025)	0.022 (0.023)	0.002
Main clinical presentations						
With	3.0 (5.5)	6.6 (8.2)	<0.001	0.042 (0.021)	0.025 (0.025)	0.001
Without	3.9 (10.3)	5.2 (6.8)	0.98	0.038 (0.029)	0.022 (0.023)	0.026
Preoperative dysfunction						
With	3.8 (6.4)	7.1 (8.1)	0.003	0.038 (0.017)	0.022 (0.024)	<0.001
Without	3.0 (2.6)	5.8 (6.3)	0.265	0.052 (0.046)	0.034 (0.019)	0.046
Pituitary adenoma type						
Lactotroph adenoma	3.4 (6.0)	6.6 (7.7)	0.023	0.035 (0.018)	0.026 (0.027)	0.068
Corticotroph adenoma	2.9 (0.9)	6.4 (5.1)	0.061	0.055 (0.033)	0.030 (0.022)	0.338
Somatotroph adenoma	5.3 (6.4)	5.8 (8.2)	0.339	0.044 (0.019)	0.027 (0.019)	0.056
Non-functional adenoma	3.8 (5.7)	7.5 (13.0)	0.055	0.05 (0.028)	0.022 (0.019)	0.001
High-risk adenomas						
Yes	2.3 (2.0)	7.2 (12.1)	0.002	0.042 (0.030)	0.021 (0.019)	0.002
No	4.3 (6.1)	6.0 (7.4)	0.07	0.036 (0.024)	0.026 (0.026)	0.006
Maximum diameter						
<2.5 cm	3.4 (3.6)	5.9 (9.3)	0.064	0.036 (0.019)	0.026 (0.020)	0.085
≥2.5 cm	3.0 (6.4)	7.2 (7.5)	0.006	0.045 (0.026)	0.021 (0.022)	<0.001
Resection degree (no. %)						
NTR	3.4 (4.0)	7.4 (7.3)	<0.0001	0.038 (0.020)	0.024 (0.018)	<0.0001
STR	4.4 (7.7)	2.4 (2.5)	0.824	0.043 (0.034)	0.041 (0.049)	0.424
Pituitary adenoma apoplexy						
Yes	2.5 (6.2)	8.0 (8.6)	0.024	0.035 (0.017)	0.023 (0.022)	0.027
No	3.8 (5.6)	5.9 (8.5)	0.027	0.042 (0.023)	0.025 (0.024)	0.001
Hardy classification						
Grades A, B	4.1 (5.5)	6.9 (7.6)	0.036	0.036 (0.017)	0.023 (0.022)	0.003
Grades C, D, E	1.5 (4.0)	5.6 (10.8)	0.004	0.050 (0.025)	0.027 (0.026)	0.006
Knosp classification (no. %)						
Grades 0, 1, 2	4.1 (5.3)	7.8 (7.7)	0.009	0.035 (0.023)	0.023 (0.020)	0.001
Grades 3, 4	1.5 (3.0)	5.0 (4.0)	0.022	0.046 (0.017)	0.034 (0.027)	0.014

p-Values were used to show the statistical difference between the age group younger than 20 years and the age group older than 20 years. Mann–Whitney test and t-test were applied.

IHC, immunohistochemistry; TILs, tumor-infiltrating lymphocytes; IQR, interquartile range; MOD, mean optical density; NTR, near total resection; STR, subtotal resection.

PD-L1 expression in the pediatric and adolescent group (0.040 (0.022)) was significantly higher than that in the >20 years age group (0.024 (0.024), p < 0.0001). Furthermore, after Bonferroni correction, PD-L1 expression level in the pediatric and adolescent group was significantly higher than that in the 21–40 years group (0.027 (0.026), p = 0.017) and >40 years group (0.023 (0.026), p = 0.017). However, PD-L1 expression was not significantly different between the 21–40 years and >40 years groups (p = 0.905; **Figures 1B–D, F**).

The entire data were divided into multiple groups based on age and clinical characteristics. In most subgroups, the CD8^+^ TILs levels were lower in the pediatric and adolescent groups but higher in the older age groups, while the expression of PD-L1 was higher in the pediatric and adolescent groups but lower in the older age group (**Table 2**).

**Figure 2:**
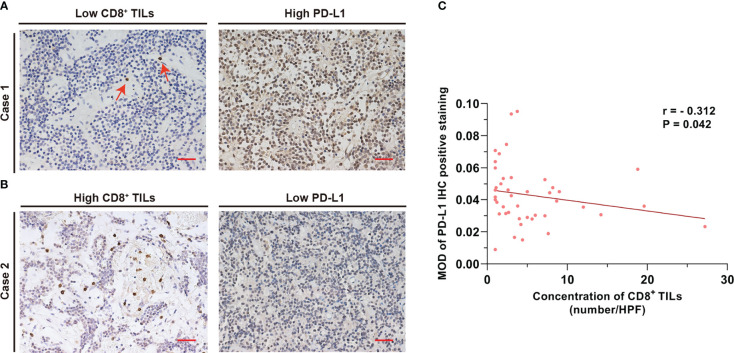
CD8^+^ TILs level was negatively associated with PD-L1 expression. **(A)** Case 1 with a low CD8^+^ TILs level but a high PD-L1 expression. The red arrow indicates CD8^+^ TILs positive staining. **(B)** Case 2 with a high CD8^+^ TILs level but a low PD-L1 expression. Scale bar, 20 µm. **(C)** The concentration of CD8^+^ TILs was negatively associated with PD-L1 level (r = −0.312, p = 0.042). TILs, tumor-infiltrating lymphocytes.

Correlation analysis revealed a negative association between CD8^+^ TILs levels and PD-L1 expression in the PAPAs group (r = −0.312, p = 0.042). In general, a higher CD8^+^ TILs level corresponded to a lower PD-L1 expression in the same case (**Figure 2**).

### CD8^+^ TILs levels and PD-L1 expression are associated with clinical features of PAPAs

**Figure 3:**
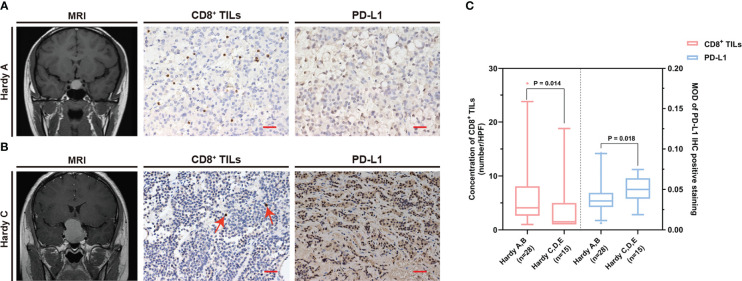
The CD8^+^ TILs levels and PD-L1 expression were associated with Hardy classification. **(A, B)** Preoperative MRI and typical CD8^+^ TILs and PD-L1 staining. Scale bar, 20 µm. **(A)** Case who had Hardy A grade tumor showed a high CD8^+^ TILs level but a low PD-L1 expression. **(B)** Case who had Hardy C grade tumor showed a low CD8^+^ TILs level but a high PD-L1 expression. The red arrow indicates CD8^+^ TILs positive staining. **(C)** The CD8^+^ TILs levels and PD-L1 expression were associated with Hardy classification. TILs, tumor-infiltrating lymphocytes.

**Figure 4:**
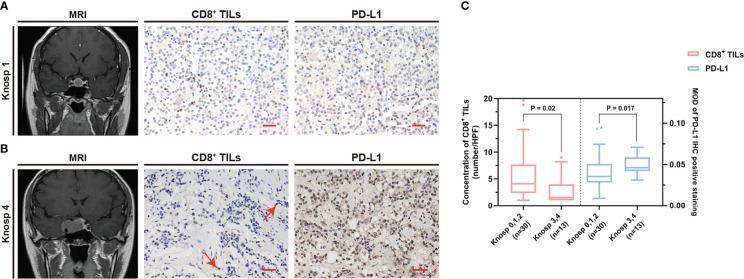
The CD8^+^ TILs levels and PD-L1 expression were associated with Knosp classification. **(A, B)** Preoperative MRI and typical CD8^+^ TILs and PD-L1 staining. Scale bar, 20 µm. **(A)** Case who had Knosp 1 grade tumor showed a high CD8^+^ TILs level but a low PD-L1 expression. **(B)** Case who had Knosp 4 grade tumor showed a low CD8^+^ TILs level but a high PD-L1 expression. The red arrow indicates CD8^+^ TILs positive staining. **(C)** The CD8^+^ TILs levels and PD-L1 expression were associated with Knosp classification. TILs, tumor-infiltrating lymphocytes.

**Figure 5:**
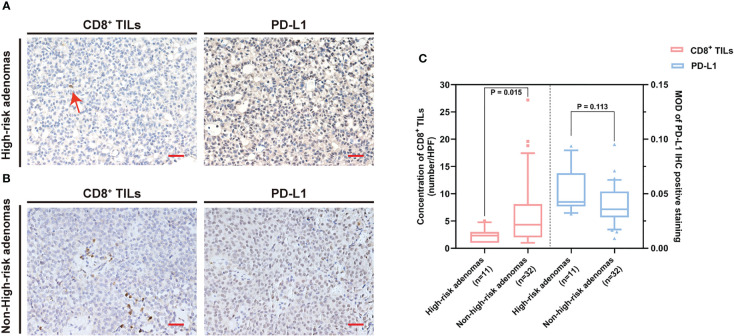
The CD8^+^ TILs levels were associated with high-risk adenomas. **(A, B)** Typical CD8^+^ TILs and PD-L1 staining of pituitary adenomas. Scale bar, 20 µm. **(A)** Case who had high-risk adenomas showed low CD8^+^ TILs. The red arrow indicates CD8^+^ TILs positive staining. **(B)** Case who had non-high-risk adenomas showed a high CD8^+^ TILs level. **(C)** The CD8^+^ TILs levels were associated with high-risk adenomas, while PD-L1 expression did not reach statistical significance. TILs, tumor-infiltrating lymphocytes.

The common clinical features of PAPAs were collected and categorized, and the association between these two biomarkers and clinical features was investigated (**Table 3**). The median value of CD8^+^ TILs level (3.4/HPF) was used to define the low (n = 21) and high (n = 22) infiltration groups, and the median value of PD-L1 level (median MOD = 0.040) was used to define the low (n = 21) and high (n = 22) PD-L1 expression groups. Statistical significance was observed with the Hardy and Knosp classification and high-risk adenomas. The CD8^+^ TILs levels were significantly higher in the Hardy A/B grade patients (n = 28, 4.1 (5.5)) than Hardy C/D/E grade patients (n = 15, 1.5 (4.0); p = 0.014; **Figure 3**), while PD-L1 expression was significantly lower in the Hardy A/B grade patients (n = 28, 0.036 (0.017)) than the Hardy C/D/E grade patients (n = 15, 0.050 (0.025); p = 0.018; **Figure 3**). The CD8^+^ TILs levels were significantly higher in the Knosp 0/1/2 grade patients (n = 30, 4.1 (5.3)) than the Knosp 3/4 grade patients (n = 13, 1.5 (3.0); p = 0.02; **Figure 4**), while PD-L1 expression was significantly lower in the Knosp 0/1/2 grade patients (n = 30, 0.035 (0.023)) than the Knosp 3/4 grade patients (n = 13, 0.046 (0.017); p = 0.017; **Figure 4**). The levels of CD8^+^ TILs were significantly lower in the high-risk adenoma patients (n = 11, 2.3 (2.0)) than in the non-high-risk adenoma patients (n = 32, 4.3 (6.1); p = 0.015; **Figure 5**). However, PD-L1 expression was not statistically significant between the above groups (p = 0.113).

**Table 3:** Correlation of CD8^+^ TILs levels and PD-L1 expression with clinical features.

Clinical feature	CD8^+^ TILs levels					PD-L1 expression				
	Quan.	p	Low	High	p	Quan.	p	Low	High	p
**Total**			21 (48.8%)	22 (51.2%)				21 (48.8%)	22 (51.2%)	
**Sex**		0.305			0.451		0.422			0.639
Male	2.7 (5.9)		11 (52.4%)	9 (40.9%)		0.041 (0.019)		9 (42.9%)	11 (50.0%)	
Female	3.8 (5.8)		10 (47.6%)	13 (59.1%)		0.036 (0.025)		12 (57.1%)	11 (50.0%)	
**Main clinical symptoms**		0.146			0.174		0.472			0.42
With	3.0 (5.5)		18 (85.7%)	15 (68.2%)		0.042 (0.021)		15 (71.4%)	18 (81.8%)	
Without	3.9 (10.3)		3 (14.3%)	7 (31.8%)		0.038 (0.029)		6 (28.6%)	4 (18.2%)	
**Preoperative dysfunction**		0.897			0.42		0.075			0.174
With	3.8 (6.4)		15 (71.4%)	18 (81.8%)		0.038 (0.017)		18 (85.7%)	15 (68.2%)	
Without	3.0 (2.6)		6 (28.6%)	4 (18.2%)		0.052 (0.046)		3 (14.3%)	7 (31.8%)	
**Pituitary adenoma type**		0.42			0.792		0.161			0.709
Lactotroph adenoma	2.7 (4.5)		12 (57.1%)	11 (50.0%)		0.036 (0.018)		13 (61.9%)	10 (45.4%)	
Corticotroph adenoma	3.0 (2.3)		3 (14.3%)	2 (9.1%)		0.071 (0.059)		2 (9.5%)	3 (13.6%)	
Somatotroph adenoma	3.5 (4.2)		2 (9.5%)	2 (9.1%)		0.035 (0.045)		2 (9.5%)	2 (9.1%)	
Non-functional adenoma	7.2 (7.0)		4 (19.1%)	7 (31.8%)		0.045 (0.022)		4 (19.0%)	7 (31.8%)	
**High-risk adenomas**		0.015			0.011		0.113			0.097
Yes	2.3 (2.0)		9 (42.9%)	2 (9.1%)		0.042 (0.030)		3 (14.3%)	8 (36.4%)	
No	4.3 (6.1)		12 (57.1%)	20 (90.9%)		0.036 (0.024)		18 (85.7%)	14 (63.6%)	
**Classification based on size**		0.294			0.329		0.283			0.223
Microadenomas	1.5 (0.7)		2 (9.5%)	0		0.022 (0.019)		2 (9.5%)	0	
Macroadenomas	3.8 (5.4)		17 (81.0%)	20 (90.9%)		0.040 (0.021)		18 (85.7%)	19 (86.4%)	
Giant adenomas	4.3 (15.0)		2 (9.5%)	2 (9.1%)		0.050 (0.043)		1 (4.8%)	3 (13.6%)	
**Maximum diameter**		0.51			0.887		0.173			0.091
<2.5 cm	3.4 (3.6)		11 (52.4%)	12 (54.5%)		0.036 (0.019)		14 (66.7%)	9 (40.9%)	
≥2.5 cm	3.0 (6.4)		10 (47.6%)	10 (45.5%)		0.045 (0.026)		7 (33.3%)	13 (59.1%)	
**Resection degree**		0.802			0.942		0.454			0.135
NTR	3.4 (4.0)		17 (81.0%)	18 (81.9%)		0.038 (0.020)		19 (90.5%)	16 (72.7%)	
STR	4.4 (7.7)		4 (19.0%)	4 (18.2%)		0.043 (0.034)		2 (9.5%)	6 (27.3%)	
**PA apoplexy**		0.406			0.449		0.364			0.449
Yes	2.5 (6.2)		8 (38.1%)	6 (27.3%)		0.035 (0.017)		8 (38.1%)	6 (27.3%)	
No	3.8 (5.6)		13 (61.9%)	16 (72.7%)		0.042 (0.023)		13 (61.9%)	16 (72.7%)	
**Hardy classification**		0.014			0.019		0.018			0.033
Grades A, B	4.1 (5.5)		10 (47.6%)	18 (81.8%)		0.036 (0.017)		17 (81.0%)	11 (50.0%)	
Grades C, D, E	1.5 (4.0)		11 (52.4%)	4 (18.2%)		0.050 (0.025)		4 (19.0%)	11 (50.0%)	
**Knosp classification**		0.02			0.015		0.017			<0.001
Grades 0, 1, 2	4.1 (5.3)		11 (52.4%)	19 (86.4%)		0.035 (0.023)		20 (95.2%)	10 (45.5%)	
Grades 3, 4	1.5 (3.0)		10 (47.6%)	3 (13.6%)		0.046 (0.017)		1 (4.8%)	12 (54.5%)	
**Recurrence**		0.0001			0.001		0.014			0.002
Yes	1.0 (0.4)		8 (38.1%)	0 (0%)		0.050 (0.025)		0 (0%)	14 (63.6%)	
No	4.2 (5.2)		13 (61.9%)	22 (100%)		0.036 (0.018)		21 (100%)	8 (36.4%)	

NTR, near total resection; STR, subtotal resection; TILs, tumor-infiltrating lymphocytes; PAs, pituitary adenomas.

Quan, quantitative data were shown as median (IQR).

The CD8^+^ TILs and PD-L1 levels were not significantly associated with sex, clinical symptoms, preoperative dysfunction, PAPAs subtypes, microadenomas, macroadenomas, giant adenomas, tumor maximum diameter, tumor resection degree, and pituitary adenoma apoplexy.

### The association between CD8^+^ TILs levels, PD-L1 expression, and PAs recurrence

**Figure 6:**
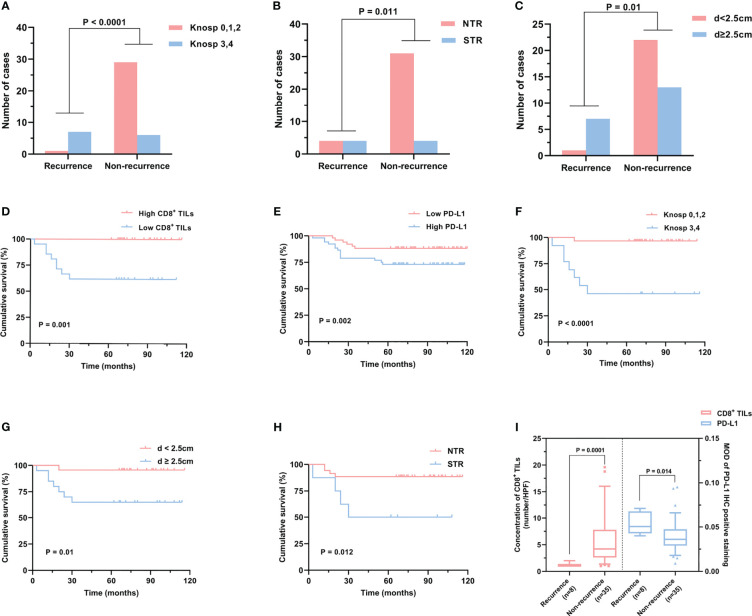
Risk factors related to PAPAs recurrence. **(A–C)** PAPAs recurrence was significantly correlated with Knosp classification **(A)**, tumor resection degree **(B)**, and tumor maximum diameter **(C)**. **(D–H)** Cumulative survival curves showed the effect of CD8^+^ TILs **(D)** and PD-L1 levels **(E)**, Knosp classification **(F)**, tumor maximum diameter **(G)**, and tumor resection degree **(H)** on PAPAs recurrence. **(I)** CD8^+^ TILs levels and PD-L1 expression showed statistically significant differences in the recurrence and non-recurrence groups. PAPAs, pediatric and adolescent pituitary adenomas; TILs, tumor-infiltrating lymphocytes.

To investigate the prognostic indicators related to PAPAs recurrence, a total of 43 PAPAs cases were divided into the recurrence group (n = 8, 18.6%) and the non-recurrence group (n = 35, 81.4%, **Table 4**). After the Mann–Whitney test, both CD8^+^ TILs (p = 0.0001) and PD-L1 (p = 0.014) levels showed statistically significant differences between the recurrence and non-recurrence groups (**Figure 6I**). Furthermore, the Knosp classification (p < 0.0001), tumor resection degree (p = 0.011), and tumor maximum diameter (qualitative data, p = 0.01) showed a statistically significant difference between the tumor recurrence and non-recurrence groups (**Figures 6A–C**). In the Kaplan–Meier analysis, CD8^+^ TILs levels (p = 0.001), PD-L1 expression (p = 0.002), Knosp classification (p < 0.0001), tumor maximum diameters (p = 0.01), and tumor resection degree (p = 0.012) were significantly associated with PFS (**Figures 6D–H**). Multivariate Cox analyses were further performed, with CD8^+^ TILs level, PD-L1 expression, high-risk adenomas, tumor maximum diameters, Hardy and Knosp classification, and tumor resection degree included. The result indicated that CD8^+^ TILs level (HR = 0.047, 95% CI 0.003–0.632, p = 0.021) was independently associated with PFS (**Table 5**). Although high-risk adenomas did not reach statistical significance in the above analysis, there was a tendency that such clinically distinct adenomas to be associated with tumor recurrence (chi-square test, p = 0.079; Kaplan–Meier analysis, p = 0.086; multivariate Cox analysis, p = 0.08).

**Table 4:** Clinical and IHC results of PAPAs recurrence.

	Recurrence	No recurrence	P	P	
**Total (no. %)**	8 (18.6%)	35 (81.4%)			
**CD8^+^ TILs (no. median (IQR))**	1.0 (0.4)	4.2 (5.2)	0.0001	
**PD-L1 (MOD, median (IQR))**	0.050 (0.025)	0.036 (0.018)	0.014	
**Sex (no. %)**				0.315
Male	5 (62.5%)	15 (42.9%)		
Female	3 (37.5%)	20 (57.1%)		
**Main clinical symptoms (no. %)**				0.084
With	8 (100%)	25 (71.4%)		
Without	0 (0%)	10 (28.6%)		
**Preoperative dysfunction (no. %)**				0.084
With	8 (100%)	25 (71.4%)		
Without	0 (0%)	10 (28.6%)		
**Pituitary tumor type (no. %)**				0.488
Lactotroph adenoma	6 (75.0%)	17 (48.6%)		
Corticotroph adenoma	1 (12.5%)	4 (11.4%)		
Somatotroph adenoma	0	4 (11.4%)		
Non-functional adenoma	1 (12.5%)	10 (28.6%)		
**High-risk adenomas (no. %)**				0.079
Yes	4 (50.0%)	7 (20.0%)		
No	4 (50.0%)	28 (80.0%)		
**Classification based on size (no. %)**				0.202
Microadenomas (<1 cm)	0	2 (5.7%)		
Macroadenomas (1–4 cm)	6 (75.0%)	31 (88.6%)		
Giant adenomas (>4 cm)	2 (25.0%)	2 (5.7%)		
**Maximum diameter (cm, median (IQR))**	3.20 (1.43)	1.86 (1.25)	0.003	
**Maximum diameter (no. %)**				0.01
<2.5 cm	1 (12.5%)	22 (62.9%)		
≥2.5 cm	7 (87.5%)	13 (37.1%)		
**Resection degree (no. %)**				0.011
NTR	4 (50.0%)	31 (88.6%)		
STR	4 (50.0%)	4 (11.4%)		
**Pituitary adenoma apoplexy (no. %)**				0.243
Yes	4 (50.0%)	10 (28.6%)		
No	4 (50.0%)	25 (71.4%)		
**Hardy classification (no. %)**				0.069
Grades A, B	3 (37.5%)	25 (71.4%)		
Grades C, D, E	5 (62.5%)	10 (28.6%)		
**Knosp classification (no. %)**				<0.0001
Grades 0, 1, 2	1 (12.5%)	29 (82.9%)		
Grades 3, 4	7 (87.5%)	6 (17.1%)		

p-Values were used to show the statistical difference between the recurrence and no recurrence groups.

IQR, interquartile range; NTR, near total resection; STR, subtotal resection; MOD, mean optical density; IHC, immunohistochemistry; PAPAs, pediatric and adolescent pituitary adenomas; TILs, tumor-infiltrating lymphocytes.

**Table 5:** Univariate and multivariate Cox analyses of different prognostic parameters for PAs recurrence.

	Univariate Cox analysis			Multivariate Cox analysis		
	P	HR	95% CI	P	HR	95% CI
**CD8^+^ TILs**	0.026	0.137	0.024–0.784	0.021	0.047	0.003–0.632
**PD-L1**	0.085	–	–	0.067	–	–
**High-risk adenomas**	0.106	–	–	0.08	–	–
**Maximum diameter**	0.011	1.72	1.13–2.60	0.135	–	–
**Hardy classification**	0.095	–	–	0.112	–	–
**Knosp classification**	0.004	21.55	2.64–175.89	0.055	–	–
**Resection degree**	0.024	4.923	1.23–19.72	0.072	–	–

PAs, pituitary adenomas; TILs, tumor-infiltrating lymphocytes.

### The expression levels of CD8 and PD-L1 mRNA in pituitary adenoma tissues

We randomly selected five cases each from the high and low CD8^+^ TILs IHC infiltration groups. We also randomly selected five cases each from the high and low PD-L1 IHC expression groups. We then quantified the expression of mRNA coding for CD8 and PD-L1 (**Supplementary Figure**). The mRNA levels of CD8 and PD-L1 were consistent with the IHC quantification. This validated the accuracy of our results for the detection of CD8 and PD-L1 proteins by IHC.

## Discussion

Pituitary adenomas are quite rare in childhood and adolescence, and patients in this age group have unique clinical characteristics and present some challenges in clinical practice when compared with adult individuals (2–5). TILs play critical roles in the development of tumors and in regulating the patient’s response to immunotherapy. In malignancies, CD8^+^ TILs have been reported to be associated with favorable prognosis (13–15). PD-L1 is an immunosuppressive factor in tumors, and anti-PD-L1 therapy has been shown to benefit many cancer patients (16, 19, 20). CD8^+^ TILs and PD-L1 have been reported to be expressed in the TME of PAs (9–11, 17, 18). However, there are few studies evaluating the expression of these biomarkers in PAPAs.

In our work, as compared with those in the aged group, the CD8^+^ TILs level was found to be significantly lower and the PD-L1 expression level was significantly higher in the PAPAs group. However, neither CD8^+^ TILs level nor PD-L1 level showed significant differences between the 21–40 years and >40 years age groups. To the best of our knowledge, few of the published studies included a relatively large PAPAs cohort to investigate the TME components, and the current study reports such an age-related difference in PAs for the first time. Remarkably, such age-related differences were found only in the TME of PA patients, while such differences were not observed at the systemic level. A decreased level of CD8^+^ TILs was also observed in the TME of younger melanoma patients. Kugel et al. reported that younger melanoma patients had lower CD8^+^ TILs levels in the TME and showed poorer therapeutic response to anti-PD1 therapy when compared with older melanoma patients. These results were also validated in a mouse model of melanoma. Furthermore, a lower ratio of CD8^+^ TILs/regulatory cells (Tregs) was also found in the TME of younger mice (30). A negative correlation between CD8^+^ TILs and Treg levels has been reported in the TME of PAs patients (31). The Treg population has been shown to change with age (32). A published study reported that Tregs induced the upregulation of PD-L1 and significantly suppressed the activity of CD8^+^ TILs (33). Therefore, in PAs, the age-related differences in CD8^+^ TILs and PD-L1 levels may be mediated by Tregs, which needs to be examined in greater depth by future studies.

Upon further analysis, we found the level of CD8^+^ TILs to be negatively correlated with the level of PD-L1 (r = −0.312, p = 0.042). CD8^+^ TILs are known to exert immune-promoting effects, whereas PD-L1 induces immune resistance by suppressing the effect of CD8^+^ TILs (34, 35). Given the opposing immunological effects of these two biomarkers, it is unsurprising to find a negative correlation between their expression in the TME.

Most PAs are characterized as non-invasive with slow growth and are circumscribed in the sellar region. However, approximately 25%–55% of PAs are invasive and infiltrate the surrounding tissues (36, 37). The Hardy and Knosp classification was utilized to assess tumor invasion into the suprasellar and parasellar regions, respectively, and Hardy C/D/E and Knosp 3/4 grades were considered invasive (38–40). The 2017 WHO classification of pituitary tumors defined high-risk adenomas as displaying distinct behavior, being clinically aggressive, invading the surrounding brain structures, growing fast, and recurring frequently, causing major challenges in their treatment (6, 41). In our study, patients with Hardy C/D/E grade coincided with a lower level of CD8^+^ TILs (p = 0.014) but a higher PD-L1 expression (p = 0.018) when compared with patients with Hardy A/B grade. Moreover, patients with Knosp 3/4 grade coincided with a lower level of CD8^+^ TILs (p = 0.02) but a higher PD-L1 expression (p = 0.017) when compared with patients with Knosp 0/1/2 grade. In addition, we found that the high-risk adenomas exhibited a lower CD8^+^ TILs level when compared with non-high-risk adenomas (p = 0.015). According to published studies, a lower CD8^+^ TILs level (11, 42) and a high PD-L1 expression (18) are associated with invasive PAs, which is consistent with our findings. CD8^+^ TILs are considered immune-promoting effectors in the TME, while PD-L1 is an immunosuppressive factor (34, 35). Additionally, cytotoxic T cells play an important role in IL-12-induced anti-angiogenic effects (43). Angiogenesis is known to be necessary for tumor growth and invasion. These data suggest that CD8^+^ TILs may inhibit the invasion of PAs, and the inhibitory effects of PD-L1 on CD8^+^ TILs may suppress CD8^+^ TILs levels in invasive PAs.

Although PAs are typically benign, their recurrence rate is approximately 20%–30% after apparent gross total resection (44). In the present work, low CD8^+^ TILs level, high PD-L1 expression, Knosp 3/4 grade, high-risk adenomas, large tumor size, and tumor subtotal resection were identified as the factors associated with tumor recurrence in PAPAs patients. Furthermore, multivariate Cox analyses revealed that CD8^+^ TILs level was an independent prognostic factor for PAPAs recurrence, which indicated the importance of CD8^+^ TILs level in the TME and its impact on the progression of PAPAs. However, the exact molecular mechanism deserves further investigation.

Our work showed that PAPAs patients had significantly different CD8^+^ TILs and PD-L1 levels in the TME when compared with adult PAs patients. Such distinct TME may influence the biological behavior of PAPAs and result in the manifestation of different clinical characteristics in comparison with adult PAs patients. Immunotherapies based on CD8^+^ TILs and PD-L1 targeted strategies have shown therapeutic effects in an increasing number of cancer patients and offer a promising prospect in PAs as well (45, 46). Furthermore, because the pituitary gland lies outside the blood–brain barrier, systemic medications may be more effective. According to certain studies, infusion of *in vitro* cultured TILs back into cancer patients had obvious tumoricidal effects (47). Additionally, ICIs including PD-1/PD-L1 axis blockade may offer a promising therapeutic strategy for treating refractory pituitary tumors, and several clinical trials have been reported in a series of isolated cases (48–51), a clinical trial with 4 cases (52), and another cohort with 15 cases (53). Although ICIs offer new treatment options for refractory pituitary tumors, endocrine dysfunction such as hypophysitis following the use of ICIs is remarkable; in this aspect, ICIs should be used with extreme caution (54). Nevertheless, it has been demonstrated that the quantity of CD8^+^ TILs and PD-L1 has a definite impact on the efficacy of tumor immunotherapy (19). Therefore, differences in the levels of CD8^+^ TILs and PD-L1 among the different age group patients may have major clinical implications when administering immunotherapy for treating PAs.

## Conclusions

PAPAs have significantly different CD8^+^ TILs and PD-L1 levels in their TME as compared with adult PAs. CD8^+^ TILs and PD-L1 levels are associated with clinical features in PAPAs. We propose that the distinct TME may influence the biological characteristics of PAPAs and result in the development of unique clinical characteristics.

## Limitations

This is a retrospective study. Due to the rarity of PAs in pediatric and adolescent subjects, the number of cases enrolled was rather limited. Thus, further studies with larger sample sizes should be performed.

## Data availability statement

The raw data supporting the conclusions of this article will be made available by the authors, without undue reservation.

## Ethics statement

The studies involving human participants were reviewed and approved by The First Hospital of China Medical University institutional review board. Written informed consent to participate in this study was provided by the participants’ legal guardian/next of kin. Written informed consent was obtained from the individual(s), and minor(s)’ legal guardian/next of kin, for the publication of any potentially identifiable images or data included in this article.

## Author contributions

MS, YS, and YZ cooperated to complete the experiment. MS, YS, YZ, and LL contributed to the collection and analysis of data. MS and YZ participated in drafting the text and figures. SH, AH, and JY designed the study and gave indispensable guidance in drafting the manuscript. All authors contributed to the article and approved the submitted version.
