# Supplementary figures and images for: PD-L1 and tumor-infiltrating CD8+ lymphocytes are correlated with clinical characteristics in pediatric and adolescent pituitary adenomas
Source: Front Endocrinol (Lausanne). 2023 Jun 22;14:1151714. doi: 10.3389/fendo.2023.1151714 (PMC10323746; doi:10.3389/fendo.2023.1151714)

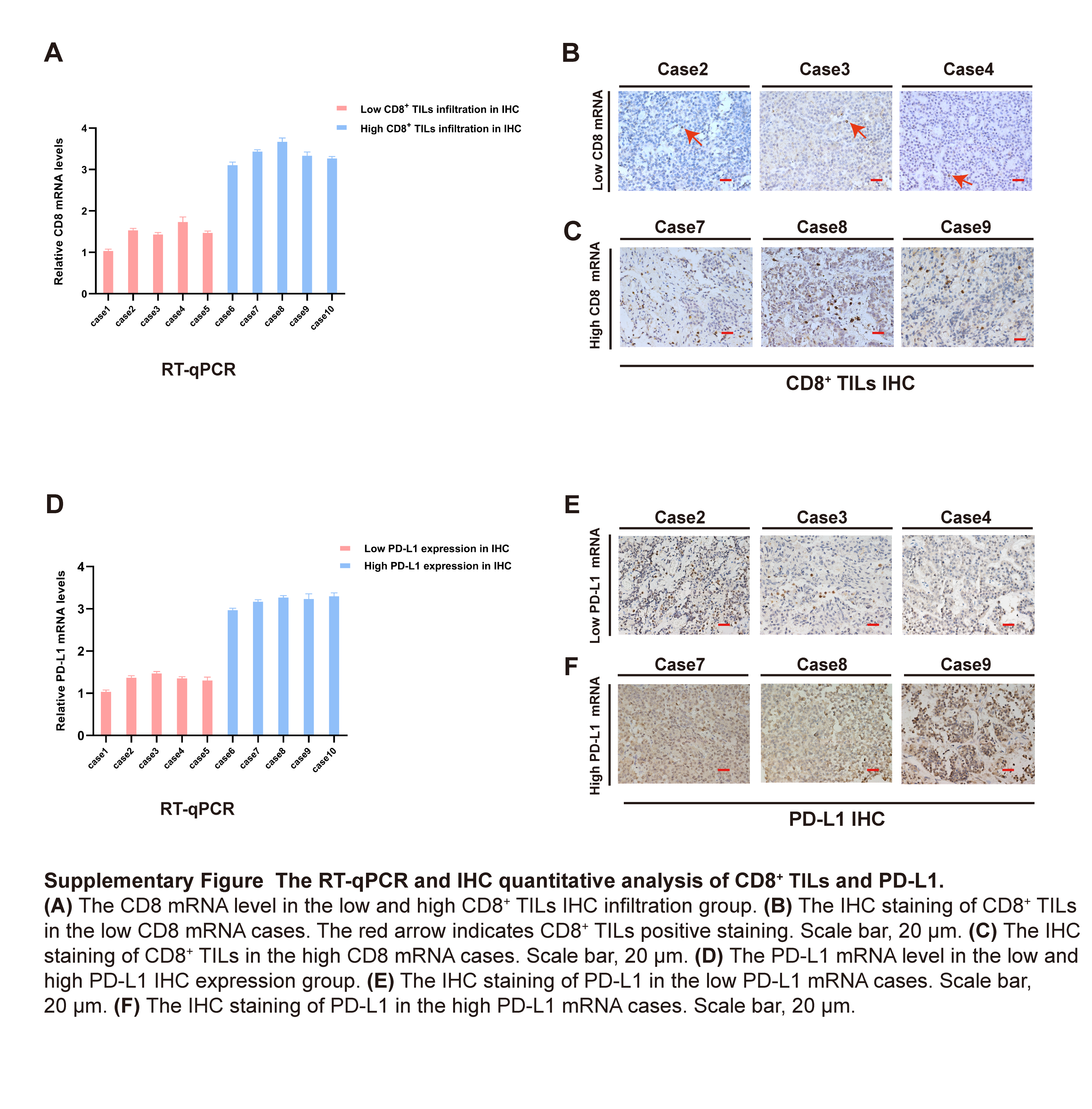

Supplement: Supplementary file 1 [file Image_1.tif]
