# Supplementary material for: PD-L1 and tumor-infiltrating CD8+ lymphocytes are correlated with clinical characteristics in pediatric and adolescent pituitary adenomas
Source: Front Endocrinol (Lausanne). 2023 Jun 22;14:1151714. doi: 10.3389/fendo.2023.1151714 (PMC10323746; doi:10.3389/fendo.2023.1151714)
Supplement: Supplementary file 2 [file Table_1.docx]

RT-qPCR primers used to measure mRNA expression (human *CD8*, *PD-L1*, and *GAPDH*).

| human |  | Primers |
| --- | --- | --- |
| *CD8* | Forward | 5'-ATGGCCTTACCAGTGACCG |
|  | Reverse | 5'-AGGTTCCAGGTCCGATCCAG |
| *PD-L1* | Forward | 5'-TGGCATTTGCTGAACGCATTT |
|  | Reverse | 5'-TGCAGCCAGGTCTAATTGTTTT |
| *GAPDH* | Forward | 5'-GAAGGTGAAGGTCGGAGTCA |
|  | Reverse | 5'-GAAGATGGTGATGGGATTTC |
